# Evaluation of Food Security in Different Grain Functional Areas in China Based on the Entropy Weight Extended Matter Element Model

**DOI:** 10.3390/foods14071111

**Published:** 2025-03-23

**Authors:** Yidi Wang, Xianzhao Liu, Mengru Song, Chenxi Dou

**Affiliations:** School of Earth Sciences and Spatial Information Engineering, Hunan University of Science and Technology, Xiangtan 411201, China; wangyidi@mail.hnust.edu.cn (Y.W.); songmengru@mail.hnust.edu.cn (M.S.); douchenxi@mail.hnust.edu.cn (C.D.)

**Keywords:** functional food areas, food security, entropy weight extended matter element model, obstacle degree

## Abstract

An accurate assessment of food security and its challenges is essential for formulating effective measures and promoting sustainable socioeconomic development. This study develops an evaluation system for China’s food security, focusing on four dimensions: food supply, food access, food production stability, and food continuity. The entropy weight extended matter element model is used for quantitative processing, which ensures that the integrity of the information can be guaranteed to a greater extent while reducing the influence of subjective factors, and then, the study evaluates the food security of different functional areas in China, and finally, it diagnoses the main obstacles to food security by using the obstacle degree model. (1) From 2000 to 2020, China’s food security level fluctuated, initially declining, and then increasing. The food security level in major production and marketing areas is generally higher, while the primary marketing areas exhibit the lowest security levels. (2) The level of grain yields and the total power of machinery per unit area are the key factors affecting food security; the impact of inputs of agricultural materials (fertilizers and pesticides) on food security has decreased and is constantly stabilizing. In the main marketing area, the per capita food share is significantly lower than in the other functional areas, which has the greatest impact on food security. (3) Analysis of the obstacle factors reveals that the food supply and access security subsystems are crucial for ensuring national, production, and marketing security. From 2000 to 2020, the average obstacle degrees of food supply and food access security at the national level were 46.56% and 21.17%, respectively; for the production and marketing areas, they were 58.47% and 21.45%; and for primary marketing areas, they were 37.69% and 26.59%. In major grain-producing areas, the main obstacles lay within the food supply security and food production stability subsystems, with average obstacle degrees of 53.77% and 15.67%, respectively, from 2000 to 2020. The above results provide a scientific basis for comprehensively improving the level of food security in China, optimizing the structure of food production in each functional area, and formulating regionalized policies for stabilizing and maintaining food production and supply.

## 1. Introduction

The people are the country’s foundation, and grains are the life of the people. As a large country with a large population, China’s food security has a bearing on the country’s fortunes and livelihood. China’s food security is facing a serious challenge as the country’s demand for food grows with the improvement of its population’s standard of living and changes in its consumption structure, coupled with the risk of production fluctuations in the agricultural cultivation process and uneven inputs of agricultural materials. Document No. 1 of the Central Committee in 2025 put forward the implementation of the grain yield improvement project, vigorously carrying out green high yield and high efficiency and stabilizing the grain sowing area and other actions. It re-emphasized that “promoting the construction of national food security industrial belt” should not be delayed and declared “basic self-sufficiency of grains and absolute safety of food rations” as the new concept of food security, and it has subsequently developed a series of innovations in food security theory and practice, which have made an important contribution to the maintenance of food security in China and the world. However, with the implementation of the “dual-carbon” goal, there is a conflict between increased agricultural production and carbon sequestration and emissions reductions, as well as the policies of the relevant authorities, which may exacerbate the risk to food security. Therefore, it is of great practical significance to carry out a comprehensive evaluation of food security in different functional grain areas in China under the background of “dual-carbon” to ensure the security and stability of China and the world.

At present, scholars at home and abroad have performed a lot of research on food security evaluation indicators and food security evaluation methods. In terms of the evaluation index system, most scholars select indicators from the aspects of food supplies, accessibility, utilization, and stability [1,2,3,4] to evaluate food security. For example, Coates [5] developed a Food Security Assessment System (FSAS) in terms of food sufficiency, nutritional adequacy, cultural acceptability, and food stability. Premanandh [6] explored the drivers affecting global food security such as population growth, climate change, and food availability, accessibility, and loss, showing that food security not only is related to food production capacity but also is closely linked to environmental sustainability. Cole [7] conducted research based on a systems perspective, combining food security with agricultural productivity, food safety, health and nutrition, and processing and supply-chain efficiency, and found that the key to ensuring food security is to achieve food and nutrition security through technological innovations in the context of social, market, and global trends. But in empirical studies on food security, commonly used indicators tend to focus on the dimensions of food access and supply, while less attention has been paid to the ability of individuals to effectively utilize accessed food to meet their nutritional needs versus whether the food supply is continuous and stable over time [8].

In China, most of the research on food security is centered on food production, mainly including the impact of land use change on food security [9,10], analysis of factors affecting food security [11,12], and changes in the spatial and temporal patterns of food security at the national and regional levels [13,14]. Some scholars have also analyzed the indicators from the perspectives of the current status of the food supply, the level of access to food, and the stability and continuity of production at the national level and the main grain-producing areas [15,16,17]. For example, Hua et al. [18] analyzed the effectiveness and problems of supply-side structural reforms on food security at the county scale in the main grain-producing areas based on synergistic theory and field research. The results show that despite the progress made in factor allocation, it still faces issues such as limited space for grain reduction, surging demand in major marketing areas, insufficient economic power in the grain-producing counties, and constrained technological potential. Lu et al. [19] found through their research that investment in overseas arable land can utilize international cooperation and food imports to have an impact on China’s food supply security, and found that when the rate of food imports exceeds 75.3%, it has a significant impact on food security. Wang et al. [20] analyzed the role of fertilizer application in guaranteeing food security in China and measured the elasticity and contribution of fertilizer application to grain yield using a regression model, and the results showed that fertilizer is an important factor affecting the increase in grain yield. Wang used the difference-in-differences method to examine the impact of the policy of the main grain-producing areas on guaranteeing national food security and further analyzed the mechanism of its impact. Lee [21] analyzed the impacts of climate change on food security in China at the provincial level and found that agricultural policies can mitigate such impacts by reducing production costs and increasing farmers’ risk resistance and motivation [22].

In terms of food security evaluation methods, currently, the commonly used evaluation methods include the TOPSIS distance function method [23,24], the weighting function method [25], the Framework for Participatory Impact Assessment (FoPIA) [26], and the DEA model [27,28]. Among them, the Topsis distance function method is highly interpretable and can clearly show the scores and rankings of food security under different indicators, which is easy to understand and compare. The weighting function method can be adapted to different evaluation needs by adjusting the weights according to the actual situation. However, these two methods are highly sensitive to the weights of the indicators, and if the weights are not reasonably allocated, it is very easy to lead to biased results. The entropy weight extended matter element model objectively determines the weights through the entropy weight method, and by combining with the theory of topable sets, it can dynamically deal with the complex relationship between the indicators and improve the stability of the evaluation. Although FoPIA combines local knowledge and experience to enhance the accuracy and usefulness of the evaluation, the results of the evaluation may be affected by the subjective opinions of the participants and require professional guidance and coordination [29]. In contrast, the entropy weight extended matter element model reduces the influence of subjective factors on the results through quantitative processing. The DEA model is capable of eliminating the effects of the input and output indicator scales and allows for comprehensive comparisons and evaluations of indicators from different units, but there are some limitations in dealing with negative outputs and the selection of input–output indicators. The entropy weight extended matter element model, on the other hand, is compatible with multiple types of data and effectively solves the system incompatibility problem. Compared with the above methods, the entropy weight extended matter element model has more advantages in improving the accuracy of food security evaluation. This method not only can maintain the integrity of information to a greater extent when integrating information [30], but also effectively solves the incompatibility problem between systems [31]; in the model, construction is relatively simple, through the correlation of the table set, it can deal with complex problems, especially suitable for the quantitative processing of multi-factor data [32], and it is widely used in many evaluation fields such as land ecological security evaluation and land use multifunctionality diagnosis [33,34].

In summary, although domestic and international scholars have conducted a great deal of research on food security, there is still room for improvement and expansion. As far as evaluation indicators are concerned, the existing research has mostly been carried out from the dimensions of food supply, accessibility, production stability, and sustainability indicators and lacks systematic and comprehensive analysis; there are also problems with diversified evaluation systems and non-uniform evaluation criteria. As far as evaluation methods are concerned, there is a certain degree of subjectivity in the assignment of weights to the indicators, which leads to a certain degree of uncertainty in the evaluation results and makes it difficult to truly reflect the state of food security [24]. In terms of research regions, most scholars choose countries, provinces, or counties as assessment units, and they mostly focus on the security situation in the main grain-producing areas, with little analysis of food security and its obstacles in grain-producing and marketing areas and in the main marketing areas. Although the contribution of grain-producing and marketing regions and main marketing regions in food security may not be as significant as that of the main producing regions, they play an equally important role in maintaining food security stability and supporting national development [25,35]. This paper evaluates the food security of different grain functional areas based on the entropy weight extended matter element model in terms of grain supply, access, production stability, and production continuity and diagnoses their key influencing factors to provide the scientific basis for improving China’s food security level. Compared with previous studies, the main contributions of this paper are in the following three aspects: (1) This study constructs an indicator system from the four dimensions of food supply security, access security, production stability, and continuity, which makes up for the lack of attention to food utilization and stability in existing studies. Consideration of agricultural production continuity (e.g., use of agricultural film, pesticides, and fertilizers) is added in light of China’s actual situation. (2) This paper adopts the entropy weight extended matter element model, which objectively determines the weights through the entropy weight method and combines with the theory of extensible sets to deal with complex problems, overcoming the limitations of traditional methods. The model maintains integrity in information integration, is suitable for the quantitative processing of multi-indicator data, and improves the accuracy and reliability of the evaluation. (3) This paper systematically evaluates food security from the dimensions of different food functional zones in China, which makes up for the shortcomings of existing studies in analyzing from a regional perspective.

## 2. Theoretical Foundations

Food security is a multidimensional concept that was first proposed by the Food and Agriculture Organization (FAO) of the United Nations in 1974. It is defined as “all people, at all times, having physical and economic access to sufficient, safe, and nutritious food that meets their dietary needs and food preferences for an active and healthy life.” With the threats of globalization and climate change, the concept of food security has gradually expanded to encompass multiple dimensions, including food supply, access, utilization, and stability. The core of food security lies in the sustainability of the food supply while ensuring a balance between economic, social, and environmental factors. The continuous evolution of this concept has deepened global awareness of food security issues. Particularly under the pressures of resource constraints, climate change, and population growth, food security has become a crucial component of global sustainable development.

In China, food security is not only essential for economic development and social stability, but is also closely linked to national strategic security and ecological civilization construction. At present, China’s agricultural development has entered a new stage, facing the dual pressures of tightening resource and environmental constraints as well as the increasing complexity of food production tasks. Especially under the “dual carbon” goals, the food production sector urgently needs to address the challenge of increasing productivity while effectively controlling carbon emissions. This necessitates a strategic focus on transforming and upgrading agricultural production methods, strengthening agricultural technological innovation, improving resource utilization efficiency, and building a food production system that balances high yield with ecological benefits. Furthermore, optimizing the food supply chain and enhancing grain reserve management mechanisms provide a solid foundation for achieving both food security and environmental protection in the “dual carbon” era. Effectively addressing this issue is not only crucial for achieving China’s national food security strategy but also a key prerequisite for promoting the green transformation of agriculture. Therefore, this study establishes a fundamental indicator system based on four dimensions: food supply security, food access security, food production stability, and food production sustainability.

Food supply security is the foundation of overall food security, as it directly determines food availability. Its key evaluation indicators include grain yield per unit area, arable land ratio, and mechanical power per unit area. Grain yield per unit area serves as a crucial measure of regional land productivity—the higher the yield, the stronger the country’s ability to ensure food security. The arable land ratio represents a fundamental resource for food production, and its decline directly weakens food production capacity, thereby posing a threat to food supply security. Meanwhile, mechanical power per unit area reflects the level of agricultural mechanization and production efficiency, both of which have a direct impact on food security. Assessing these indicators provides a comprehensive understanding of food supply capacity and serves as a scientific basis for formulating food production policies. These indicators align with the “availability” dimension of the food security framework proposed by the Food and Agriculture Organization [24].

Food access security focuses on whether individuals or households can obtain sufficient food through purchasing, production, or other means. It is closely linked to socioeconomic development levels, with food access challenges being particularly prominent in poverty-stricken areas. A key evaluation indicator of food access security is per capita food availability, which serves as an essential measure of food accessibility. A higher per capita food availability suggests a more equitable distribution of food, ensuring that basic population needs are met. Assessing this indicator helps identify inequalities in food access and provides a basis for formulating food distribution policies to enhance food security. This indicator aligns with the “affordability” dimension of the Global Food Security Index but places greater emphasis on regional food distribution patterns [23].

Food production stability refers to the ability of the food production system to withstand external shocks, such as natural disasters, climate change, and economic fluctuations, to ensure a continuous and reliable food supply. Key evaluation indicators include the food production fluctuation coefficient and affected farmland area, which help identify risk factors in food production. Assessing these indicators provides a scientific basis for formulating risk management policies to mitigate potential threats. This dimension aligns with the “stability” aspect of the FAO food security framework.

Food production sustainability focuses on the efficiency of resource utilization and the environmental impact of food production, ensuring its long-term viability. As a critical dimension in food security evaluation, it assesses the sustainability of agricultural practices over time. Key evaluation indicators include fertilizer, pesticide, and agricultural film usage as well as diesel consumption, which helps identify environmental issues within food production. Analyzing these indicators provides a scientific basis for formulating sustainable agricultural policies. This dimension aligns with relevant Sustainable Development Goals but places a more specific emphasis on agricultural production practices.

## 3. Materials and Methods

### 3.1. Study Area

**Figure 1:**
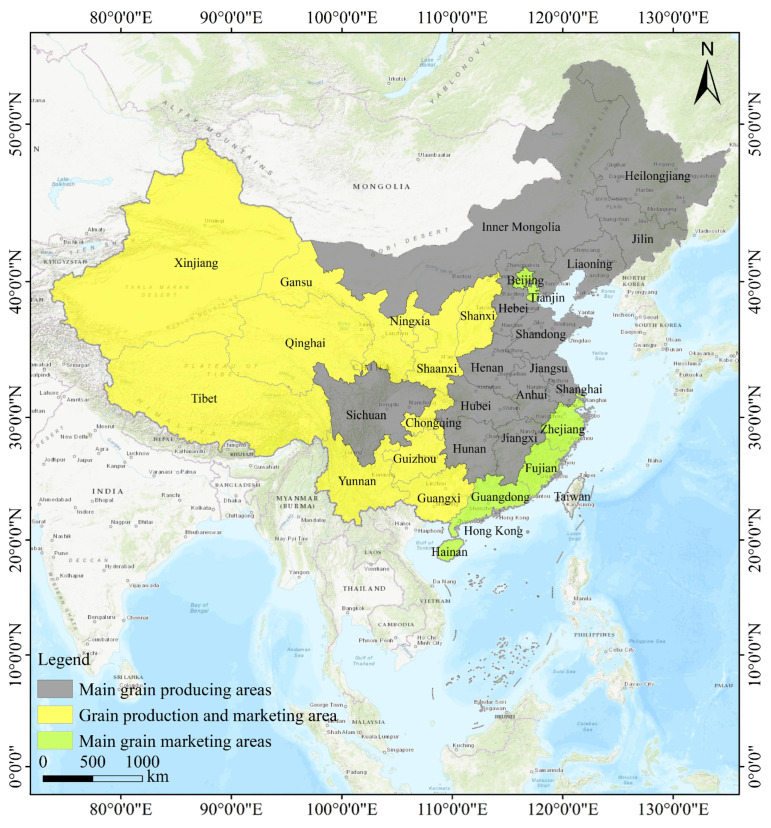
The spatial distribution of China’s three major grain functional areas.

According to the Guiding Opinions on the Establishment of Functional Grain Production Areas and Important Agricultural Product Production Protection Areas issued by the State Council in 2017, the Chinese mainland is divided into three functional grain areas, namely, the main grain production area, the grain production and marketing balance area, and the main grain marketing area, by comprehensively taking into account the economic situation, agricultural cultivation capacity, resource conditions, as well as the production and consumption of grains of the provinces (autonomous regions and municipalities directly under the central government) (Figure 1). Specifically, the main grain-producing areas are concentrated in 13 provinces in the central and northeastern parts of the country, such as Henan, Shandong, Hebei, Anhui, Jiangsu, Jilin, and Heilongjiang. Most of the provinces have a favorable temperate monsoon climate, fertile soil, and abundant water resources, which are extremely suitable for the extensive cultivation of grain crops and have high potential for grain production. The main production areas are not only able to achieve self-sufficiency in food production, but also to provide adequate food support for other regions, and they are the core areas of the national food supply. Grain-producing and marketing areas are located mainly in the western and northwestern provinces of Xinjiang, Inner Mongolia, Ningxia, Gansu, and Qinghai, where, because of the constraints imposed by natural conditions such as topography and climate, the land is relatively decentralized, and grain production is able to satisfy only local demand, making a relatively small contribution to the country’s total grain output. The main grain marketing areas, on the other hand, are concentrated in the southeastern coastal provinces with rapid economic development, including Guangdong, Fujian, Zhejiang, Shanghai, Hainan, Beijing, and Tianjin, where the reduction in the area of arable land as a result of the rapid development of urbanization and industrialization, coupled with large populations and limited land resources, has led to a large gap between food production and demand in the region.

### 3.2. Construction of Food Security Indicator System

Based on the connotation and theoretical foundations of food security, and in order to comprehensively describe China’s food security problems as much as possible, the authors draw on the existing research results; follow the principles of scientificity, systematization, objectivity, comparability, and operability; select 10-factor indicators from the four dimensions of food supply, access, production stability, and production sustainability to construct China’s food security evaluation index system; and analyze and evaluate food security and its change trend in 31 provinces in China. The specific indicators are shown in Table 1.

### 3.3. Entropy Weight Extended Matter Element Model

The meta-model of topable objects is a multi-factor evaluation method for solving incompatible and complex problems that is able to quantitatively measure the topologizability of things and their level of synthesis on the basis of ensuring the integrity of integrated information [34,36]. In view of the complexity of food security evaluation and the incompatibility between the selected indicators, this paper adopts the entropy weight extended matter element model to systematically and comprehensively evaluate food security, and its main steps are as follows [37,38].

#### 3.3.1. Determination of Food Security Object Elements

Assuming that the food security *N* has *n* features $c_{1},c_{2}\ldots c_{n}$ and corresponding quantities $v_{1},v_{2}\ldots v_{n}$, the food security object element can be expressed as follows:

(1) $$M=\left[\begin{array}{ccc} N & c_{1} & v_{1}\\ & \vdots & \vdots \\ & c_{n} & v_{n} \end{array}\right]$$

where *M* is the *n*-dimensional food security material element, abbreviated M=(N,c,v). In order to effectively evaluate the level of food security in China, the article categorizes the results of food security evaluation into five levels: I (insecure), II (critically secure), III (basically secure), IV (relatively secure), and V (secure).

#### 3.3.2. Classical Domain, Section Domain, and Object Element to Be Evaluated Determination

First, the classical domain object element matrix for food security can be expressed as

(2) $$M_{oj}=\left(N_{oj},c_{i},v_{o}\right)=\left[\begin{array}{ccc} N_{oj} & c_{1} & \left(a_{oj1},b_{oj1}\right)\\ & \vdots & \vdots \\ & c_{n} & \left(a_{ojn},b_{ojn}\right) \end{array}\right],$$

where $M_{oj}$ is the classical domain object element; $N_{oj}$ is the jth evaluation level of food security classification; $c_{i}$ is the ith evaluation indicator; and $\left(a_{ojn},b_{ojn}\right)$ is the range of quantitative values corresponding to evaluation level j, i.e., the classical domain. The class interval of the classical domain is clearly defined using isometric splitting.

Second, the node domain object element matrix for food security can be expressed as

(3) $$M_{p}=\left(N_{p},c_{i},v_{p}\right)=\left[\begin{array}{ccc} N_{p} & c_{1} & \left(a_{p1},b_{p1}\right)\\ & \vdots & \vdots \\ & c_{n} & \left(a_{pn},b_{pn}\right) \end{array}\right],$$

where $M_{p}$ is the nodal domain object element, the specific value of the section is determined based on the overall range of values of the food security evaluation indicator classification standard; $v_{p}=(a_{p1},b_{p1})$ is the range of quantitative values of the node domain object element with respect to the feature $c_{i}$; $v_{i}$ is the characteristic value corresponding to the $c_{i}$; and p is for all levels of food security.

Finally, determine the object element to be evaluated, $M_{x}$, and denote the object element of the object to be evaluated, $N_{x}$, as

(4) $$M_{x}=\left[\begin{array}{ccc} N_{x} & c_{1} & v_{1}\\ & \vdots & \vdots \\ & c_{n} & v_{n} \end{array}\right].$$

In the formula, the letters have the same meaning as before.

#### 3.3.3. Determining the Correlation Function

The food security indicator correlation function $K\left(x_{i}\right)$ is defined as

(5) $$K\left(x_{i}\right)=\left\{\begin{array}{c} -\frac{\rho \left(X,X_{O}\right)}{\left|X_{O}\right|},\;\;\;\;X\in X_{O}\\ \frac{\rho \left(X,X_{O}\right)}{\rho \left(X,X_{P}\right)-\rho \left(X,X_{O}\right)},\;\;\;\;X\notin X_{O} \end{array}\right.,$$

where $\rho \left(X,X_{O}\right)$ is the distance of the point X from the finite interval $X_{O}=\left[a_{O},b_{O}\right]$; $\rho \left(X,X_{P}\right)$ is the distance of the point X from the finite interval $X_{p}=\left[a_{p},b_{p}\right]$; $\left|X_{O}\right|=\left|b_{O}-a_{O}\right|$, X, $X_{O}$, and $X_{p}$ are the magnitude ranges of the food security elements to be evaluated, the magnitude ranges of the classical domain elements, and the range of the nodal domain object element, respectively. Then, $\rho \left(X,X_{O}\right)$ and $\rho \left(X,X_{P}\right)$ are expressed, respectively, as follows:

(6) $$\rho \left(X,X_{O}\right)=\left|X-\frac{1}{2}(a_{O}+b_{O})\right|-\frac{1}{2}(b_{O}-a_{O})$$

(7) $$\rho \left(X,X_{P}\right)=\left|X-\frac{1}{2}(a_{p}+b_{p})\right|-\frac{1}{2}(b_{p}-a_{p}).$$

#### 3.3.4. Evaluation Level Determination

The food security evaluation grade was determined by calculating the comprehensive correlation degree and the eigenvalues of the grade variables.

(1) Calculate the composite relevance and determine the evaluation level. The composite correlation $K_{j}\left(N_{x}\right)$ of the object to be evaluated $N_{x}$ with respect to rank j is

(8) $$K_{j}\left(N_{x}\right)=\sum_{i}w_{ij}K_{j}\left(x_{i}\right)$$

(9) $$K_{jx}=max\left\{K_{j}\left(N_{x}\right)\right\}\left(j=1,2,3,\ldots ,m\right),$$

where $K_{j}\left(x_{i}\right)$ is the single-indicator correlation of the object to be evaluated, $N_{x}$, with respect to the rank j; $w_{ij}$ is the weight of each evaluation indicator; $K_{jx}$ indicates that the object to be evaluated, $N_{x}$, belongs to food security criterion level j, where, if $K_{ji}=max\left\{K_{j}\left(x_{i}\right)\right\}$, (*j* = 1, 2, 3, ……, *m*), then the *i*th indicator of the object to be evaluated belongs to the food security standard level *j*.

(2) Calculate the appraisal eigenvalues:

(10) $$\bar{K_{j}}\left(N_{x}\right)=\frac{K_{j}\left(N_{x}\right)-minK_{j}\left(N_{x}\right)}{maxK_{j}\left(N_{x}\right)-minK_{j}\left(N_{x}\right)}$$

(11) $$j^{*}=\frac{\sum_{j=1}^{m}j\bar{K_{j}}\left(N_{x}\right)}{\sum_{j=1}^{m}\bar{K_{j}}\left(N_{x}\right)}$$

where $\bar{K_{j}}\left(N_{x}\right)$ is the average of the composite relevance $K_{j}\left(N_{x}\right)$ of the object to be evaluated, $N_{x}$, with respect to the rank j; $j^{*}$ is the eigenvalue of the rank variable, which is used to measure the level of the object element in the high, medium, and low levels. Within the same class, the smaller the eigenvalue, the higher the level of food security, and the larger the eigenvalue, the lower the level of food security (Table 2). Food security assessment is more accurate when $K_{jx}$ is combined with $j^{*}$. It is assumed that the food security level is n, and $j^{*}$ belongs to the interval $n^{\prime }$; when n = $n^{\prime }$, food security is at the medium level of n; if $n^{\prime }$ = n−1, then food security is a high level in the n-level; if $n^{\prime }$ = n+1, then food security is a low level in the *n*-level.

#### 3.3.5. Weighting of Evaluation Indicators

The improved entropy method was used to determine the indicator weights.

First, the data are normalized and the formula is calculated as follows:

(12) $$\mathrm{Positive}\;\mathrm{indicators}:\;x_{ij}^{\prime }=\frac{x_{ij}-{min}_{ij}}{{max}_{ij}-{min}_{ij}}$$

(13) $$\mathrm{Negative}\;\mathrm{indicators}:\;x_{ij}^{\prime }=\frac{{max}_{ij}-x_{ij}}{{max}_{ij}-{min}_{ij}}$$

where $x_{ij}$ is the observed value of food security evaluation indicator *i* in year *j*; ${max}_{ij},{min}_{ij}$ are the maximum and minimum values of indicator *i* during the study period; and $x_{ij}^{\prime }$ is the normalized value of $x_{ij}$, $x_{ij}^{\prime }$ ∈ [0, 1].

Second, construct the entropy decision matrix:

(14) $$P_{ij}=\frac{x_{ij}^{\prime }}{\sum_{j=1}^{n}x_{ij}^{\prime }}(j=1,2,\ldots ,n)$$

(15) $$e_{j}=-\frac{1}{lnn}\sum_{j=1}^{n}p_{ij}lnp_{ij}$$

where $P_{ij}$ is the integrated standardized value and $e_{j}$ is the entropy value of the jth indicator.

Finally, calculate the indicator weights:

(16) $$W_{j}=\frac{I_{j}}{\sum_{i=1}^{n}I_{j}}(j=1,2,\ldots ,n)$$

In Equation (16), $W_{j}$ is the weight of the jth indicator, which is calculated in Table 1. $I_{j}=1-e_{j}$, which expresses the coefficient of variation of the jth indicator.

### 3.4. Obstacle Degree Model

Food security is affected by a number of factors, each of which affects it to a different degree. In order to identify the key factors affecting food security, this paper adopts the obstacle degree model for specific analysis [41], and the calculation steps are as follows.

First, calculate the weight of a single factor on the total objective, i.e., the factor contribution $F_{ij}$:

(17) $$F_{ij}=W_{j}\times P_{ij}$$

where $W_{j}$ is the weight of the jth indicator, and $P_{ij}$ is the weight of the ith region under the jth indicator in this indicator.

Second, calculate the gap between the single-factor indicator and the regional food security index, i.e., the indicator deviation $I_{ij}$:

(18) $$I_{ij}=1-R_{ij}^{\prime }$$

where $R_{ij}^{\prime }$ is the value of the indicator calculated after normalization.

Finally, calculate the evaluation indicator obstacle degree $H_{ij}$:

(19) $$H_{ij}=\frac{F_{ij}\times I_{ij}}{\sum_{j=1}^{n}F_{ij}\times I_{ij}}$$

In the formula, $H_{ij}$ is the obstacle degree of the indicator, and the larger its value is, the greater the impact of the indicator on the results; at the same time, we can obtain the dimensional obstacle degree $U_{ij}$ by summing up $H_{ij}$.

### 3.5. Data Sources

Taking the provincial area of mainland China as the basic unit, the data on arable land area, provincial area, disaster-affected area, agricultural machinery power, fertilizer, pesticide, pesticide film, diesel fuel use, effective irrigated area, sown area of grain crops, and grain output required for calculating the food security indicators come from the China Rural Statistical Yearbook for the period of 2001–2021, the China Statistical Yearbook, and the provincial statistical yearbooks and bulletins. Hong Kong, Macao, and Taiwan were not included in this study given the availability of data. For a few provinces with missing data in certain years (e.g., agricultural film usage data for Xinjiang and Hainan in 2001 and 2005, and for Gansu in 2002 and 2006), this study applied linear interpolation to fill the missing values. Specifically, it was assumed that the missing data points had a linear relationship with their adjacent known data points, and the missing values were estimated by calculating the slope between these adjacent points.

## 4. Results

### 4.1. Analysis of Factors Affecting Food Security

As can be seen from Table 1, among the four subsystems of food security, the food supply security system has the largest weight of 0.6052, followed by food access security (0.2271), while food production sustainability security and food production stability security have smaller weights of 0.0924 and 0.0754, respectively. This suggests that food supply security plays a crucial role in maintaining China’s food security, while international concerns about food security are mainly focused on food supply, stability, and utilization efficiency [42]. The reasons for this discrepancy may be related to the specificity of China’s food security problem, i.e., China is faced with a large population and insufficient per capita resource possession, and coupled with regional differences in agricultural productivity and ecological constraints, food supply has become a bottleneck affecting China’s food security and sustainable development [43]. Therefore, when exploring China’s food security, food supply security must be given sufficient attention.

Analysis of the individual indicators of the food security subsystem reveals that four indicators, namely, total mechanical power per unit area (0.2639), per capita food possession (0.2271), the proportion of arable land area (0.1864), and the level of food yields (0.1549) have a greater impact on food security, and their combined weights account for 83.23% of the system that affects food security. This shows that China has effectively guaranteed food security by supporting agricultural development through multi-level initiatives. First of all, China’s implementation of fiscal subsidy policies, increased investment in special funds, and promotion of advanced agricultural technologies have significantly improved agricultural production efficiency and the stability of the food supply. Secondly, the dynamic optimization of arable land management through land protection policies and land reclamation measures has provided reliable land security for food production. In addition, the increase in the level of grain yields has been made possible by the popularization of good varieties, the application of new cultivation techniques, and the improvement of farmland infrastructure. Finally, the process of agricultural mechanization has been accelerated, and the efficiency and scale of grain cultivation have been substantially increased, relying on the policy of subsidizing the purchase of machinery and the mechanized service system. These measures complement each other and together build a comprehensive and solid food security guarantee system. This is basically consistent with Xu Ming’s [24] conclusion that arable land resources are the main constraint on China’s food production. However, this study found that the impact of total mechanical power per unit area on food security was significantly greater than that of arable land, suggesting that increased investment in and promotion of the total power of agricultural machinery is equally critical to guarantee national food security.

### 4.2. Evaluation of Food Security Based on the Entropy Weight Extended Matter Element Model

#### 4.2.1. Correlation and Comprehensive Evaluation Analysis of Food Security Indicators at the National Level

Based on the index data and index weights of each province in China (Table 1), the correlation degree of food security evaluation indicators in different years at the national level was calculated by using the entropy weight extensible matter element method. Taking the calculation of grain yield level (c1) in 2000 as an example, the correlation of this indicator is $K_{1}$ = −0.285, $K_{2}$ = −0.035, $K_{3}$ = 0.152, $K_{4}$ = −0.170, $K_{5}$ = −0.361, and according to the principle of the maximum value, it can be determined that this indicator belongs to the level of $K_{3}$ [44], i.e., the “III Level (basic safety)” level. The same can be derived from the specific level of other indicators in each year (Table 3).

The same is true for the calculation of the combined correlation of food security at the national level for different years. The comprehensive correlations corresponding to food security in 2000 were *K*_1_(N_2000_) = −0.397, *K*_2_(N_2000_) = −0.215, *K*_3_(N_2000_) = −0.164, *K*_4_(N_2000_) = −0.256, and *K*_5_(N_2000_) = −0.252, which leads to the conclusion that the level of China’s food security was at level $K_{3}$ in 2000 [44], i.e., “level III (basic security)”, and similarly, the specific results of food security level in other years can be derived (Table 4).

**Figure 2:**
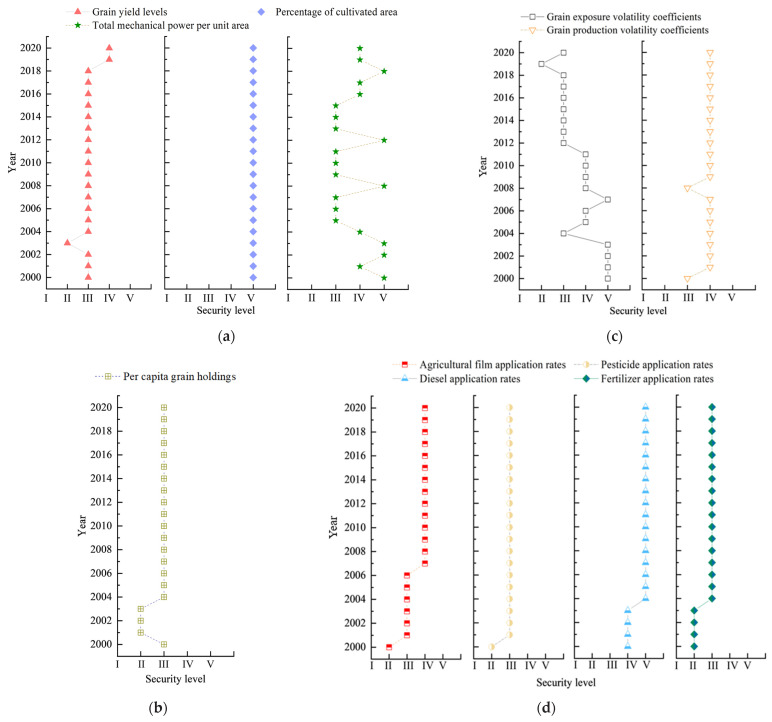
Relevance level of food security evaluation indicators at the national level from 2000 to 2020. (**a**) Food supply security; (**b**) food access security; (**c**) stability and security of food production; (**d**) sustained food production security.

As can be seen in Figure 2, the security levels of the food yield level ($c_{1}$), the proportion of arable land area ($c_{2}$), and the total mechanical power per unit area ($c_{3}$) in the food supply subsystem at the national level are significantly different over time. The rating of $c_{1}$ was at level III (basic safety) in most of the years of the study period, except for 2003, which was at level II (critical safety), and 2019–2020, which was at level IV (comparative safety), indicating that the level of food yields is more adaptable to the external environment. The safety level of $c_{2}$ is safe (V) in all years, and the remarkably high stability indicates that the percentage of cultivated area always has an intrinsic regulation mechanism to maintain it in the long term, except that it may be regulated by national policies. The more drastic changes in $c_{3}$, which varied between class III (basic safety) and class V (safety) during the study period, indicate that the total power of agricultural machinery may be more sensitive to innovations or contingency responses [45]. In the food access subsystem, per capita grain holdings ($c_{4}$) was at level II (critical security) in 2001–2003 and level III (basic security) for the remaining years, a trend that reflects the effectiveness of China’s food security policies and the important role of technological progress in agriculture. The early fluctuation of the per capita food possession security level is related to the agricultural tax reform, the minimum purchase price pilot, and the adjustment of the grain circulation system reform policy, while its continued “basic security” state may be due to technological innovation and the improvement of production efficiency, and the overall small fluctuation of the Chinese population during the study period may also be an important reason.

In the grain production stability subsystem, the rank of the grain exposure volatility coefficients ($c_{5}$) showed a fluctuating downward trend, which may be related to the increase in the frequency of agricultural planting disasters and the intensification of climate fluctuations in recent years [46]. The safety level of grain production volatility coefficients, on the other hand, fluctuates less, with the exception of 2000 and 2008, when it was at level III (basically safe) status, and the rest of the years were at level IV (relatively safe) status. In the food production continuity subsystem, on the other hand, the safety levels of agricultural film application rates ($c_{7}$), diesel application rates ($c_{9}$), and fertilizer application rates ($c_{10}$) showed an increasing trend in 2000–2020 and have been in a stable state since 2004 (agricultural film application rates have been in a steady state since 2007), reflecting the rapid advancement of agricultural modernization and national policy support [47]. In addition, the safety level of pesticide application rates ($c_{8}$) has remained relatively stable, and except for 2000, when it was at level II (critically safe), it was at level III (basically safe) in all other years, indicating that the use of pesticides has been restricted under the promotion of agro-environmental protection and green agriculture and that their safety levels have tended to stabilize or improve.

**Figure 3:**
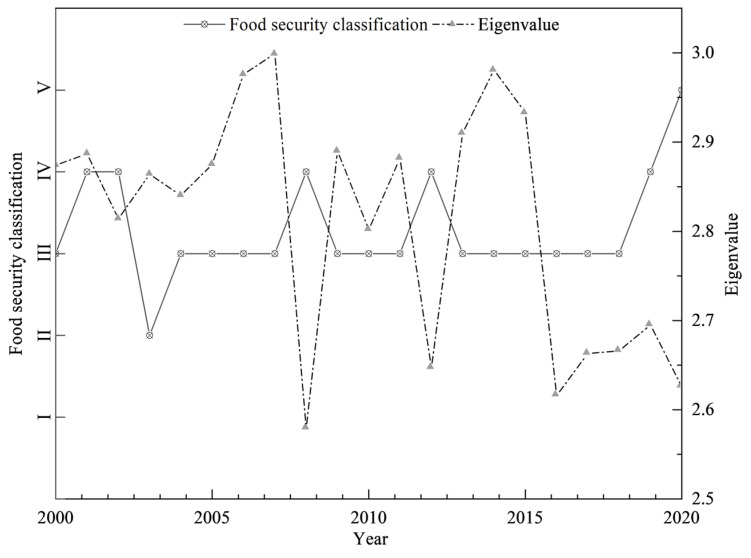
Food security levels and characteristic values in China from 2000 to 2020.

Figure 3 shows the temporal evolution of China’s food security level from 2000 to 2020. Overall, the national level of food security has shown a fluctuating upward trend, rising from level II (critical safety) in 2003 to level V (safety) in 2020, with most years reaching level III (basic safety) or above. In 2020, the $j^{*}$ value of food security reached 2.626, which is at a moderate level in the V category. This suggests that China’s food security level was increasing in 2000–2020 and that the grain yield levels, percentage of cultivated area, grain production volatility coefficients, and per capita grain holdings, as well as the grain production sustainability security subsystems, were the main drivers of the increase in grain production volatility levels in China (Figure 2). In terms of time periods, changes in China’s food security rating can be roughly divided into three stages, with the first stage (2000–2004) being the fluctuation stage. During this period, the food security correlation between grain production volatility coefficients of agricultural film application rates and pesticide application rates was improved in 2001–2002 due to the influence of policies such as “direct grain subsidies and agricultural tax reform”, and the correlation levels were raised to III and IV, respectively (Figure 2d). In 2003, the correlation level of grain yield levels of important indicators dropped from Grade III to Grade II, which led to a decrease in the food security level, but the value of food security $j^{*}$ was 2.864, so it belonged to the medium level of Grade II, which indicated that the decrease in the food security level in 2003 might have been affected by unexpected events, and it was only a temporary decrease in the food security level; in 2004, the correlation level of both grain yield levels and per capita food possession was raised to Grade III. In 2004, the correlation between the level of grain yields and per capita grain holdings were both raised to level III. The years 2005–2018 is the second phase. The food security level was relatively stable in most of the years in this phase, and the analysis of the $j^{*}$ values found that the food security level in this phase was medium level III in most of the years, except for 2008 and 2012, when it was level IV. During this period, the safety level of total mechanical power per unit area correlation changed most significantly, i.e., fluctuating between level III and level V (Figure 2), which may be related to the “Agricultural Machinery Purchase Subsidies” implemented by the state during this period. The third stage is 2019–2020, in which the state put forward Opinions on Effectively Strengthening the Construction of High-standard Farmland to Enhance the National Food Security Guarantee Capability, emphasizing the hiding of grain on land and technology, with the primary goal of enhancing grain production capacity, which led to a steady increase in grain yield levels and a rise in the correlation with food security to IV, which in turn led to a significant increase in China’s level of food security (Figure 3).

#### 4.2.2. Analysis of the Correlation and Comprehensive Evaluation of Food Security Indicators in Different Functional Areas

**Figure 4:**
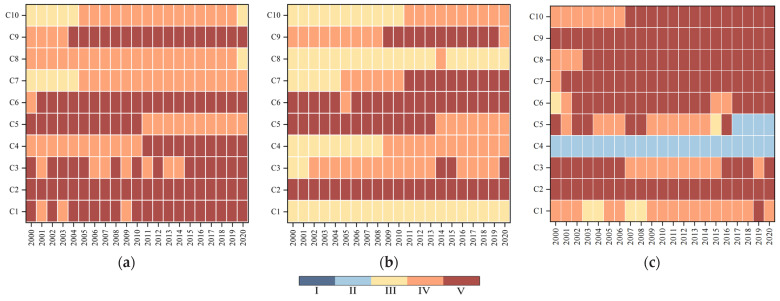
Heat map of food security evaluation indicator levels for different food functional areas in 2000–2020. (**a**) Main grain producing areas; (**b**) grain production and marketing area; (**c**) main grain marketing areas.

According to the data of each indicator of food security evaluation in each provincial area and the weights of the indicators, the correlation and the comprehensive correlation rank of the food security evaluation indicators of different food functional areas were obtained by using the aforementioned topological objective meta-model. As can be seen in Figure 4, in the grain supply subsystem, the grain yield levels ($c_{1}$) and total mechanical power per unit area ($c_{3}$) in both the main grain-producing areas and the main marketing areas show a fluctuating trend, and the fluctuating years are mainly concentrated in the vicinity of 2002–2008. This may be due to the fact that the total grain production in the main producing and marketing areas continued to decline during this period, reaching a historical low of 3.06 × 10^8^t and 3.42 × 10^7^t, respectively, in 2003. In order to cope with the risk of food shortage, the state introduced a series of policies to support and benefit agriculture, such as exemption of agricultural tax, direct food subsidies, comprehensive agricultural subsidies, etc., and the safety level of $c_{1}$ and $c_{3}$ indicators was ensured [48]. On the other hand, in the three major grain functional areas, the percentage of the cultivated area has always maintained a “safe” level, which is related to the country’s determination to adhere to the red line of 1.8 billion mu of arable land and ensure food security. From the analysis of the food access subsystem, it was concluded that the security level of per capita grain holdings ($c_{4}$) in both the main grain-producing areas and the production and marketing areas gradually increased, with a level escalation occurring in 2009–2010, mainly due to the impact of the continuous decline in the state of grain production in 2000–2003, where the decline in grain production exposed the shortcomings of policy security and agricultural production efficiency, which triggered the country’s heightened attention to the issue of food security. Subsequently, the government introduced a series of strong policies, including increasing financial inputs, implementing a minimum purchase price policy for grain, strengthening the promotion of agricultural science and technology, and increasing agricultural subsidies, as a result of which the per capita grain holdings have continued to rise. In contrast, the demand for food in the main food marketing areas is much higher than the food production capacity, owing to the high population density and developed economy. The shortage of grain leads to a need to rely heavily on the allocation of the main grain-producing areas, and the main grain-producing areas are greatly affected by natural disasters or climate change, so the food supply cannot fully meet the demand [49], resulting in the correlation level of per capita grain occupancy in the main selling areas being always in a state of “critical security”.

From the grain production stability subsystem, the rank of the grain exposure volatility coefficients ($c_{5}$) between the main grain-producing areas and the production and marketing areas showed a downward trend in 2010–2015 that has since leveled off. The rank of the grain exposure volatility coefficients in the main grain marketing areas showed a fluctuating decrease, which is directly related to the fact that the main marketing areas are located in the eastern coastal areas that are affected by natural disasters such as typhoons and storm surges. The rank changes in grain production volatility coefficients ($c_{6}$) in the three major grain functional zones are relatively stable, with fluctuations occurring only in individual years, indicating that the agricultural production planning of each functional zone is more reasonable, and the control ability to withstand the fluctuation of grain production and the risk of food supply is stronger [50]. In terms of the food production continuity subsystem, since the main food marketing areas are not directly responsible for large-scale food production, indicators such as agricultural film and pesticide application rates are mostly at the “safe” level. The main grain-producing areas and the production and marketing areas, both of which bear the responsibility of food production security, saw an increase in the rank of each indicator around 2004 due to the impact of agricultural subsidy policies, the promotion of intensive production methods, the reduction in the number of agricultural material inputs, etc. [51].

**Figure 5:**
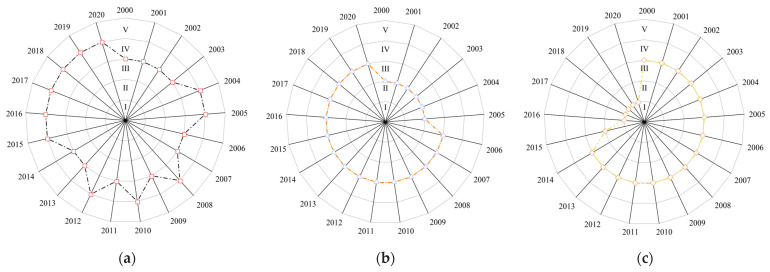
Radar map of food security evaluation ratings of different food functional zones in 2000–2020. (**a**) Main grain producing areas; (**b**) grain production and marketing areas; (**c**) main grain marketing areas.

In terms of the level of food security in the different functional areas (Figure 5), the food security level of the main grain-producing areas in 2000–2020 is maintained at level III or above, which is closely related to the higher food supply security and food access security (per capita grain holdings) levels of the region. However, during this period, there were certain fluctuations in the food security level of the main producing areas from 2003 to 2015, and the analysis of relevant indicators shows that the grain yield level and the total mechanical power per unit area played a decisive role in the change in food security level in the region (Figure 4). The analysis of grain production and marketing areas shows that the grain security level in 2000–2020 showed an upward trend over time and has been stabilized at level III since 2006, which may be related to the change in the correlation level of the production volatility coefficients in 2006 that prompted the improvement in the grain security level in the production and marketing areas. Although the correlation of the indicators fluctuated in other years, the mutual constraints between multiple indicators guaranteed the stability of food security in the production and marketing areas. In 2000–2020, the food security level of the main marketing area was on a downward trend; food security was at the level of III in 2000–2014, but the food security level began to decline in 2015 and stabilized at the level of I in 2016–2020. The reason for this is the fragile foundation of food production, circulation, and consumption security in the main marketing areas, the weak capacity of their own food production and security, supply security, and the limited capacity of regulation and emergency response security, which leads to the continued low level of food security [52]. Therefore, it is imperative to accelerate the construction of a high-quality, high-efficiency green food security systems in the main marketing areas.

### 4.3. Diagnosis of Food Security Disorder Factors

According to the aforementioned diagnostic model of obstacle degree, the top five obstacle factors affecting China’s food security from 2000 to 2020 and their obstacle degree magnitude can be obtained.

Considering the space limitation, Table 5 shows only the results for the years 2000, 2010, and 2020. As can be seen from Table 5, the sum of the obstacle degrees of the top 5 obstacle factors affecting food security at the national level is 91.72%, 83.07%, and 78.06% in 2000, 2010, and 2020, respectively. The top three obstacle factors are always grain yield levels, total mechanical power per unit area, and per capita grain holdings, and the sum of the obstacle degrees of the three factors in 2000, 2010, and 2020 is 62.42%, 67.38%, and 65.81%, respectively, which implies that the security of grain supply and access is the key to guaranteeing food security. That is, the security of the grain supply and the security of grain access are China’s food security, i.e., the security of the food supply and the security of food access are the two main obstacle subsystems to food security in China. The obstacle degree of the food supply security subsystem to China’s food security is on the rise, while the obstacle degree of the food access security subsystem is on the decline. However, in terms of individual factors, the obstacle degree of grain levels yield and per capita grain holdings have been decreasing over time, while the obstacle degree of total mechanical power per unit area has been increasing, which indicates that with the popularization and application of modern agricultural technology, the efficiency of agricultural production has been increasing and the capacity of food supply has been increasing, while the further enhancement of mechanization faces resistance, so more attention needs to be paid to the intelligent enhancement of agricultural productivity and agricultural technology innovation [49,51]. In contrast, the obstacle degree of the two subsystems of food production stability security and continuity security to national food security during this period shows an obvious decreasing trend, with its obstacle degree decreasing from 19.28% and 10.02%, respectively, in 2000 to 0.00% and 4.65% in 2020, indicating that while improving the level of food security, China is also facing challenges such as limited agricultural resources, increasing difficulty in improving productivity, and how to optimize the structure of food production to meet the market demand.

From the perspective of food functional areas (Table 5), the obstacle degree of the food supply security subsystem is generally high in the three functional areas, and the average obstacle degree of the main grain production area, the production and marketing area, and the main marketing area in 2000–2020 is 53.77%, 58.48%, and 37.69%, respectively. Among them, the average obstacle degree of total mechanical power per unit area in the main production and marketing areas is 39.61% and 32.65%, respectively, while the average obstacle degree in the main marketing area is only 16.88%, and this difference suggests that the main marketing area has less resistance to the development of mechanical power, whereas the main production and marketing areas are still facing greater technical bottlenecks [53]. Additionally, the grain yield levels in each functional area have long constrained the improvement of food security, but in 2020, the grain yield levels in the main production areas did not show a significant limiting effect. This is mainly because the main production areas have effectively improved agricultural production efficiency by relying on the grain direct subsidy and agricultural machinery subsidy policies. However, the main marketing area has reduced the cultivated area due to urban expansion, and the degree of restriction of the percentage of the cultivated area has increased significantly. The policy of land use control and urban priority development has led to the continuous reduction in agricultural land in the main sales area. In addition, the imbalance in the diffusion of mechanization and intelligent technologies in different regions has further widened regional differences in the impact of policies and technologies on food security [54,55]. In terms of the food access security subsystem, the per capita grain holdings obstacle between the production and marketing areas and main marketing areas has existed for a long time, with an average obstacle of 21.46% and 26.59% from 2000 to 2020, accounting for 22.86% and 35.52% of their total obstacle, while the obstacle in main production areas has continued to decline, with an obstacle of 0 in 2020. The long-standing barriers to food access security in the production and marketing areas and the main sales areas indicate that there are problems in food allocation, market circulation, and supply chain efficiency in these regions, while the continuous decline in the barriers in the main producing areas indicates that the efficiency of food access and distribution has been greatly improved. From the subsystem of food production stability and security, the obstacles of each indicator in the three functional regions have been decreasing, and only the obstacles of the grain production volatility coefficients in the main marketing regions have increased in 2020, which may be due to the high dependence of the main marketing regions on external food, which is exacerbated by global events such as epidemics and climatic anomalies. From the subsystem of food production sustainability and security, the total obstacle degree of food production sustainability and security increased in the main production areas, mainly due to the dependence on chemical fertilizers and pesticides to increase production, which led to soil degradation and environmental problems, thus affecting the sustainability of food production [56]; the production and marketing areas have no obvious obstacle problems due to the efficient use of resources and the relative stability of production. Pesticide application rates ($c_{8}$) in the main marketing area increased in 2010, but pesticide use has gradually decreased with the promotion of green agricultural technology, reflecting its progress in environmental protection and production sustainability [57,58].

## 5. Discussion

### 5.1. Food Security Assessment

In this paper, food security at the national level and in different food functional areas was evaluated using the entropy weight topological object meta-model. The results found that the level of food security in China shows a development trend of decreasing and then increasing, which is similar to the results obtained by Zhang et al. [23] using the entropy-weight TOPSIS model. For different food functional regions, the food security level of the main production and marketing regions is higher overall, while that of the main marketing regions is lower due to their limited food supply capacity. The reason is that the food security of the main marketing regions is more dependent on the food supply from other regions, which means that under the framework of guaranteeing national food security, interregional food distribution and supply chain management need to be further optimized to cope with the risk of supply chain disruption that may occur in the future. In terms of the subsystems of food security, the food supply security subsystem has the greatest impact on overall food security, especially the higher weights of grain yield levels and total mechanical power per unit area, which corroborates the results of Xu and Chen [24]. The difference is that the weight of total mechanical power per unit area is larger in this study, but as the level of agricultural mechanization continues to increase, its contribution to food security shows a gradual slowing trend, indicating that in terms of mechanization technology, there is limited room for further improvement, and there is a need to increase investment in intelligent and precision agriculture technology. In addition, the stability of the percentage of cultivated area, as an important factor in guaranteeing food security, reflects the importance attached to the protection of cultivated land in national policies, especially the effective implementation of the policy of “1.8 billion mu of cultivated land red line” [59]. In the food access security subsystem, per capita grain holdings have a significant impact on food security. In particular, as the area of arable land decreases rigidly, the challenge of food access security becomes more acute, especially in densely populated main marketing areas. Although the state has mitigated food shortages in the main marketing areas through the food transfer policy, the stability of the food supply remains a key issue that needs to be urgently addressed in the future due to the impact of uncontrollable factors such as climate change and natural disasters [60]. The analysis of the grain production volatility coefficients of the subsystem of grain production stability shows that the coefficient of grain exposure volatility coefficients fluctuates markedly in individual years as a result of the impact of natural disasters. This reflects the fact that, in the face of global climate change, the risk management and disaster response capacity of China’s grain production still needs to be further improved in order to ensure the stability of food security. In the food production sustainability subsystem, the use of agricultural substances such as chemical fertilizers and pesticides has been reduced and stabilized, indicating that agricultural production has begun to shift toward a more environmentally friendly and sustainable direction with the advancement of the concept of green agricultural development.

In summary, the findings suggest that future policy directions should emphasize targeted technological advancements, regional differentiation strategies, and robust risk management systems. Strengthening institutional and technical capacities to cope with risks associated with global climate change and other unpredictable shocks will be crucial to maintaining China’s food security sustainably.

### 5.2. Diagnosis of Food Security Obstacles

Through the analysis of obstacle factors, it can be found that food supply security and food access security are the main obstacle factors affecting China’s food security. Among them, the level of grain yield, the total mechanical power per unit area, and the per capita grain possession are the most important obstacles, which also reflects the key role of modern agricultural technology in ensuring the food supply. Chen et al. [61] used the continuous double difference method to analyze the impact of high-standard farmland construction policy on food production capacity and its mechanism of action and found that the promotion of the level of agricultural mechanization is essential to guarantee the security of the food supply. Peng et al. [62] also found that agricultural mechanization has significantly improved grain production efficiency, but they also pointed out that the current level of mechanization development is entering a bottleneck stage and needs to be further transformed to automation and transformation. This is in line with the results of the paper, but this paper found that the level of agricultural mechanization in the main producing and marketing areas has long entered a bottleneck to further improve the level of yields in limited space. Therefore, it is necessary to promote the progress of food production technology through scientific and technological innovation and policy support to eliminate the impact of these obstacles on food security [63]. Therefore, there is a need to promote the advancement of food production technology through scientific and technological innovation and policy support in order to eliminate the impact of these obstacles on food security. In addition, the issue of food access security in the main marketing areas cannot be ignored. It is worth pointing out that factors such as global climate change and epidemics have exacerbated the uncertainty of the food supply, and there is a need to optimize the supply chain and grain reserve system in the future to ensure food security in the main marketing areas.

## 6. Conclusions and Policy Implications

In this study, a Chinese food security evaluation index system was constructed based on the four dimensions of food supply security, food access security, food production stability, and food production continuity. The entropy weight extended matter element model and obstacle degree model are used to quantitatively analyze the level of food security in different functional regions of China and systematically assess their security status, as well as to identify the main obstacle factors affecting food security. The results of the study are as follows:

(1) During the period from 2000 to 2020, China’s food security level experienced a trend of change, first declining and then rising, with an overall positive development trend. By 2020, the eigenvalue of the food security level reached 2.626, marking that it had reached the medium level of Grade V (security), reflecting the continuous improvement of China’s food security situation. The food security level in the main grain-producing areas and the production and marketing areas is high overall, while the main marketing areas have a lower security level due to their limited grain supply capacity.

(2) The level of grain yields and the total power of machinery per unit area are key factors affecting food security. As agricultural mechanization increases, its contribution to food security shows a gradual slowing trend, indicating the need for greater investment in intelligent and precision agriculture technologies. With changes in population and arable land area, food access security is increasingly challenged, especially in the densely populated main marketing areas.

(3) The food supply and access security subsystem is the key to guaranteeing food security at the national level in the food producing and marketing areas and in the main marketing areas. Among them, the average obstacles of the food supply subsystem and food access security subsystem at the national level from 2000 to 2020 are 46.56% and 21.17%, respectively; in the production and marketing areas, they are 58.47% and 21.45%; and in the main marketing areas, they are 37.69% and 26.59%. The main obstacles in the main food producing areas are concentrated in the food supply security and food production stability security subsystems, with an average obstacle degree of 53.77% and 15.67%, respectively, from 2000 to 2020.

Therefore, different food functional areas need to take into account regional characteristics, develop precise policy interventions, reduce the degree of obstacles through technological innovation, and promote the overall improvement of food security. With regard to the main production areas, the construction of food production infrastructure should be further strengthened, and the process of planning the layout and construction of high-standard farmland should be accelerated. It focuses on promoting the upgrading of agricultural mechanization, automation, and standardized equipment; accelerating the research and development and popularization and application of precision agriculture technology; and breaking through the technical barriers to traditional agricultural mechanization. At the same time, it actively promotes highly efficient green agricultural production technologies; continuously improves comprehensive food production efficiency; strictly controls and reduces the use of fertilizers, pesticides, and other agricultural inputs; responds to the goals of the national “dual-carbon” strategy; and realizes sustainable agricultural development.

With regard to the balanced production and marketing areas, it is necessary to continue to increase scientific and technological research and development and promotion, accelerate the construction of agricultural infrastructure, and improve the comprehensive production capacity and risk-resistant capacity of food. Efforts have been made to improve the construction of the grain circulation system, optimize the interregional grain logistics regulation and distribution network, improve the efficiency of cross-regional grain dispatch and market regulation and control capacity, and safeguard the dynamic balance of regional supply.

With regard to the main marketing areas, measures should be taken to expand and optimize channels for the confident supply of grain, promote the construction of a long-term cooperation mechanism with the main producing areas and areas with a balance of production and marketing, and enhance the ability to provide a stable supply of grain across the regions. At the same time, it has accelerated the construction and upgrading of automated, informatized, and modernized grain reserve warehouse facilities and established a platform for the emergency management of grain reserves through the strengthening of the regional supply risk monitoring event early warning system and emergency response mechanism to improve the comprehensive guarantee capacity to respond to market abnormalities and public health and other emergencies.

Overall, differentiated and precise policy measures should be implemented on the basis of the obstacles to food security in different functional regions, strengthening the application of agricultural scientific and technological innovations, enhancing the efficiency of the entire food industry chain, optimizing the food market supply system, and promoting the realization of China’s food security in the long term in a stable and sustainable development fashion.

Although this paper systematically analyzes the issue of food security in China, there is still room for further improvement. First, the meaning of food security is much broader than what is reflected in the current indicator system. Due to the limitation of data, it is difficult to make a comprehensive analysis of food security, and China’s food security will be further analyzed according to the structural characteristics of agricultural crops in the future; second, this study mainly uses statistical data, and the study period is 2000–2020, which cannot reflect the changes in food security in real time. Remote sensing technology combined with a support vector machine can classify and extract food crops and arable land, which not only can improve the accuracy of crop classification but also provide continuous time series information. For example, Shi, S. [64] used Sentinel-1 and Landsat-7 images to extract the average texture features of the gray level covariance matrix (GLCM) and combined them with the support vector machine to classify and extract cropland in food crop growing areas. Based on the classification results, meteorological data, and normalized vegetation index (NDVI), the annual grain yield was predicted by calculating the conversion formula of annual net primary productivity to yield. Finally, the definition of classical and nodal domains using the entropy weight extended matter element model was evaluated for the lack of accurate hierarchical standard intervals; thus, the selection of indicators and the setting of classical and nodal domain intervals still need to be further explored.

## Figures and Tables

**Table 1:** Food security evaluation indicator system and indicator weights.

Evaluation Dimension	Evaluation Indicator	Characterization of Indicators	Indicator Property	Indicator Weight
Food supply security (0.6052)	Grain yield levels (*c*_1_)	Total grain production/provincial area	+	0.1549
	Percentage of cultivated area (*c*_2_)	Cultivated land area/province area	+	0.1864
	Total mechanical power per unit area (*c*_3_)	Total power of agricultural machinery/cultivated area	+	0.2639
Food access security (0.2271)	Per capita grain holdings (*c*_4_)	Total food production/resident population in the province	+	0.2271
Stability and security of food production (0.0754)	Grain exposure volatility coefficients (*c*_5_)	Area affected/area sown with crops	−	0.0281
	Grain production volatility coefficients (*c*_6_)	$\left|\frac{Y_{t}-Y_{t}^{\prime }}{Y_{t}^{\prime }}\right|$	−	0.0473
Sustained food production security (0.0924)	Agricultural film application rates (*c*_7_)	Agricultural film application rates/crop sown to crops	−	0.0181
	Pesticide application rates (*c*_8_)	Pesticide application rates/area sown to crops	−	0.0206
	Diesel application rates (*c*_9_)	Diesel consumption/area sown to crops	−	0.0204
	Fertilizer application rates (*c*_10_)	Fertilizer application rates/area sown to crops	−	0.0333

Note: $Y_{t}$ in the coefficient of food production volatility denotes total food production in year *t*, and $Y_{t}^{\prime }$ denotes the five-year moving average of food production.

**Table 2:** $j^{*}$ classification [39,40].

Level	High	Medium	Low
$j^{*}$	<2.5	2.5–3.5	>3.5

**Table 3:** Relevance and evaluation level of China’s food security indicators in 2000, 2010, and 2020.

Indicator Correlation	2000							2010	2020
	$K_{1}$	$K_{2}$	$K_{3}$	$K_{4}$	$K_{5}$	$K_{m}$	Level	Level	Level
*K_j_*(*c*_1_)	−0.285	−0.035	0.152	−0.170	−0.361	0.152	III	III	IV
*K_j_*(*c*_2_)	−0.476	−0.457	−0.439	−0.357	0.219	0.219	V	V	V
*K_j_*(*c*_3_)	−0.478	−0.428	−0.333	−0.142	0.002	0.002	V	III	IV
*K_j_*(*c*_4_)	−0.302	−0.008	0.011	−0.412	−0.647	0.011	III	III	III
*K_j_*(*c*_5_)	−0.464	−0.423	−0.318	−0.167	0.008	0.008	V	IV	III
*K_j_*(*c*_6_)	−0.330	−0.062	−0.042	−0.080	−0.917	−0.042	III	IV	IV
*K_j_*(*c*_7_)	−0.312	0.170	−0.085	−0.390	−0.542	0.170	II	IV	IV
*K_j_*(*c*_8_)	−0.400	0.003	−0.002	−0.501	−0.701	0.003	II	III	III
*K_j_*(*c*_9_)	−0.441	−0.366	−0.135	0.370	−0.210	0.370	IV	V	V
*K_j_*(*c*_10_)	−0.293	−0.035	−0.147	−0.432	−0.574	−0.035	II	III	III

**Table 4:** Evaluation results of China’s food security rating in 2000, 2010, and 2020.

Composite Correlation	$K_{1}$	$K_{2}$	$K_{3}$	$K_{4}$	$K_{5}$	$K_{m}$	Level	j*
*K_j_*(N_2000_)	−0.397	−0.215	−0.164	−0.256	−0.252	−0.164	III	2.874
*K_j_*(N_2010_)	−0.402	−0.241	0.010	−0.190	−0.337	0.010	III	2.802
*K_j_*(N_2020_)	−0.421	−0.294	−0.058	−0.029	−0.240	−0.029	IV	2.626

**Table 5:** Obstacles to food security in China and different food functional zones and their degree of obstruction, 2000–2020.

Obstacle Factors		National Level Obstacles (%)			Obstacle Level in the Main Production Area (%)			Obstacle Level in the Production and Marketing Area (%)			Obstacle Level in the Main Marketing Area (%)		
		2000	2010	2020	2000	2010	2020	2000	2010	2020	2000	2010	2020
Food supply security	$c_{1}$	18.45	17.09	13.47	13.48	13.84	0.00	26.07	25.64	25.76	10.92	10.28	9.94
	$c_{2}$	0.00	0.00	7.60	8.11	0.00	7.05	0.00	0.00	0.00	9.40	8.75	13.15
	$c_{3}$	19.90	30.61	32.57	20.82	37.46	60.56	34.43	30.74	32.79	13.64	24.18	12.81
Food access security	$c_{4}$	24.07	19.68	19.77	21.79	13.68	0.00	20.82	20.56	22.99	27.61	23.07	29.10
Stability and security of food production	$c_{5}$	19.28	9.83	0.00	23.75	12.21	11.06	14.41	13.94	4.43	13.47	0.00	0.00
	$c_{6}$	0.00	0.00	0.00	0.00	0.00	0.00	3.36	2.01	0.00	0.00	0.00	8.28
Sustained food production security	$c_{7}$	0.00	0.00	0.00	0.00	0.00	0.00	0.00	0.00	3.64	0.00	0.00	0.00
	$c_{8}$	0.00	5.86	4.65	0.00	6.84	5.71	0.00	0.00	0.00	0.00	9.98	0.00
	$c_{9}$	10.02	0.00	0.00	0.00	0.00	0.00	0.00	0.00	0.00	0.00	0.00	0.00
	$c_{10}$	0.00	0.00	0.00	0.00	0.00	4.89	0.00	0.00	0.00	0.00	0.00	0.00
Total		91.72	83.07	78.06	87.95	84.03	89.27	99.09	92.89	89.61	75.04	76.26	73.28

## Data Availability

The original contributions presented in this study are included in the article. Further inquiries can be directed to the corresponding author.

## References

[1] Chaudhary A., Gustafson D., Mathys A. (2018). Multi-Indicator Sustainability Assessment of Global Food Systems. Nat. Commun..

[2] Hartwig T., Nguyen T.T. (2023). Local Infrastructure, Rural Households’ Resilience Capacity and Poverty: Evidence from Panel Data for Southeast Asia. JED.

[3] Magaña-Lemus D., Lara-Álvarez J., Schmitz A., Kennedy P.L., Schmitz T.G. (2015). Food Security Measurement: An Empirical Approach. Frontiers of Economics and Globalization.

[4] Owusu V., Ma W., Emuah D., Renwick A. (2021). Perceptions and Vulnerability of Farming Households to Climate Change in Three Agro-Ecological Zones of Ghana. J. Clean. Prod..

[5] Coates J. (2013). Build It Back Better: Deconstructing Food Security for Improved Measurement and Action. Glob. Food Secur..

[6] Premanandh J. (2011). Factors Affecting Food Security and Contribution of Modern Technologies in Food Sustainability. J. Sci. Food Agric..

[7] Cole M.B., Augustin M.A., Robertson M.J., Manners J.M. (2018). The Science of Food Security. NPJ Sci. Food.

[8] Manikas I., Ali B.M., Sundarakani B. (2023). A Systematic Literature Review of Indicators Measuring Food Security. Agric. Food Secur..

[9] Yuan J., Chen W., Zeng J. (2023). Spatio-temporal differentiation of cropland use change and its impact on cropland NPP in China. J. Nat. Resour..

[10] Cui G., Bai X., Wang P., Liu Y., Xu Y., Dong L. (2022). Food security impact gauged by carbon accounting of food life cycle. J. Beijing Norm. Univ. (Nat. Sci.).

[11] Yuan S., Li G. (2024). Evaluation and measurement of influencing factors of food security in China. J. Beijing Univ. Agric..

[12] Huang J. (2022). Evaluation and Influencing Factors of Food Security in the Yangtze River Economic Belt HUANG Jian-feng. J. Anhui Agric. Sci..

[13] Wang S., Wang Y., Fang Z., Lv H., Liu L. (2024). Grain eco-efficiency in China: Horizontal measurement, spatio-temporal pattern evolution and influencing factors. J. Agric. Resour. Environ..

[14] Jiang H., Chen Y., Liu Z. (2023). Spatiotemporal Pattern and Influencing Factors of Grain Production Resilience in China. Econ. Geogr..

[15] Fu Z., Cai Y., Yang Y., Dai E. (2001). Research on the relationship of cultivated land change and food security in China. J. Nat. Resour..

[16] Liu Z., Han Y. (2020). Difficulties and Solutions of Ensuring Food Supply Under the Rural Revitalization Strategy. J. Northwest AF Univ. (Soc. Sci. Ed.).

[17] Zhang Z., Li C., Jin Y., Cui Z. (2023). Rural financial exclusion, agricultural technological progress and food supply security. Sci. Res. Manag..

[18] Hua S., Zhong Y. (2019). Food supply-side reform and food security. Rural Econ..

[19] Lu X., Ke S. (2017). Research of China’s food supply security based on farmland investment overseas. China Popul. Resour. Environ..

[20] Wang Z., Xiao H. (2008). Analysis of the effect of chemical fertilizer application on the growth of food production. Issues Agric. Econ..

[21] Wang S., Wu H., Li J., Xiao Q., Li J. (2024). Assessment of the Effect of the Main Grain-Producing Areas Policy on China’s Food Security. Foods.

[22] Lee C.-C., Zeng M., Luo K. (2024). How Does Climate Change Affect Food Security? Evidence from China. Environ. Impact Assess. Rev..

[23] Zhang X., Bao J., Xu S. (2023). Research on the evaluation of China’s food security based on entropy weight TOPSIS model. Chin. J. Agric. Resour. Reg. Plan..

[24] Xu M., Chen S. (2024). Measurement and Influencing Factors of China’sGrain Security Level—Based on Panel Data from 31 Provinces from 2012 to 2022. Henan Soc. Sci..

[25] Yao C., Teng Y., Huang L. (2015). Evaluation index system construction and empirical analysis on food security in China. Trans. Chin. Soc. Agric. Eng. (Trans. CSAE).

[26] Schindler J., Graef F., König H.J., Mchau D. (2017). Developing Community-Based Food Security Criteria in Rural Tanzania. Food Sec..

[27] Wang R., Li S., Jiang Y. (2018). Comprehensive evaluation on China’s food import security based on super efficiency DEA model. Acta Agric. Zhejiangensis.

[28] Gao J., Li H., Shao J. (2020). Security assessment of China’s grain industry based on DEA model. Stat. Decis..

[29] Sun C., Zhen L., Wang C., Hu J., Du B. (2015). Biodiversity simulation of poyang lake wetlands by InVEST model under different scenarios. Resour. Environ. Yangtze Basin.

[30] Qiu L., Fan S., Ru K., Sun Q., Zhang S. (2018). Ecological Security of Cultivated Land in Qingliu County Analyzed by Entropy Matter-Element Model. Fujian J. Agric. Sci..

[31] Mou S., Yan J., Sha J., Deng S., Gao Z., Ke W., Li S. (2020). A Comprehensive Evaluation Model of Regional Water Resource Carrying Capacity: Model Development and a Case Study in Baoding, China. Water.

[32] Wong H., Hu B.Q. (2014). Application of Improved Extension Evaluation Method to Water Quality Evaluation. J. Hydrol..

[33] Guan L., Feng Z., Liu G., Shi Y., Wang J., Lin F., Ma X. (2020). Evaluation of land eco-security in Fenhe River Basin based on matter-element model. Chin. J. Ecol..

[34] Gao J., Liu Y., Zheng H. (2021). Land Use Multifunctional Diagnosis Based on Entropy Weight and Matter-element Model. Chin. J. Agric. Resour. Reg. Plan..

[35] Liu H. (2021). Guaranteeing food security can’t be focused only on the main production areas. Economic Daily.

[36] Zhao Y., Xue X., Huang Y., Kong H. (2021). Evaluating Comprehensive Carrying Capacity of Coastal Area Using the Matter-Element Extension Method: A Case Study in Fujian Province of China. Ocean Coast. Manag..

[37] Luo W., Wu C., Wang Y., Wu Y., Wu Z. (2008). Evaluation on Urban Land Ecological Level Based on Matter Element Analysis: A Case of Hangzhou City in Zhejiang Procince. China Land Sci..

[38] Huang H., Luo W., Wu C., Li D. (2010). Evaluation of land eco-security based on matter element analysis. Trans. CSAE.

[39] Liu S., Li W. (2019). Indicators Sensitivity Analysis for Environmental Engineering Geological Patterns Caused by Underground Coal Mining with Integrating Variable Weight Theory and Improved Matter-Element Extension Model. Sci. Total Environ..

[40] Wang X., Yu H., Lv P., Wang C., Zhang J., Yu J. (2019). Seepage Safety Assessment of Concrete Gravity Dam Based on Matter-Element Extension Model and FDA. Energies.

[41] Wu K. (2008). Comprehensive Evaluation on the Development of Agricultural Circular Economy in Chaohu Basin. China Popul. Resour. Environ..

[42] Varzakas T., Smaoui S. (2024). Global Food Security and Sustainability Issues: The Road to 2030 from Nutrition and Sustainable Healthy Diets to Food Systems Change. Foods.

[43] Yang G. (2020). Challenges of the Sustainable Development of Agriculture in China. Sustain. Dev..

[44] Shi K., Diao C., Zuo T., Sun X., Sun Y. (2013). Evaluation of eco-security of cultivated land requisition-compensation balance based on entropy weight and matter element model. Chin. J. Eco-Agric..

[45] Peng J., Wu H., Wang W. (2021). The influence of agricultural mechanization level on farmers’ production of staple food. Chin. J. Agric. Resour. Reg. Plan..

[46] Lu F., Xie Y. (2008). The Supply of and the Demand for Grains in China, and the Trend of Prices (1980~2007). Manag. World.

[47] Li H., Zhang W., Zhang F., Du F., Li L. (2010). Chemical fertilizer use and efficiency change of main grain crops in China. J. Plant Nutr. Fertil..

[48] Wang J., Xiao H. (2013). The nature and prospects of China’s eight-year increase in grain production. Issues Agric. Econ..

[49] Hu T., Ju Z., Liu X. (2023). Towards Sustainable Food Security through Regional Grain Supply and Demand Analysis in China. Int. J. Environ. Res. Public Health.

[50] Li J. (2024). Sustainable Agricultural Management and Ecological Environmental Protection. Hans J. Agric. Sci..

[51] Jiang B., Tang W., Li M., Yang G., Deng X., Cui L. (2023). Assessing Land Resource Carrying Capacity in China’s Main Grain-Producing Areas: Spatial–Temporal Evolution, Coupling Coordination, and Obstacle Factors. Sustainability.

[52] Yang D., Jiang H. (2017). Food Security of Main Grain Sales Regions in China—Based on a Gap in Balance between Grain Supply and Demand. J. Agric. Sci. Technol..

[53] Liu X., Li X. (2023). The Influence of Agricultural Production Mechanization on Grain Production Capacity and Efficiency. Processes.

[54] Liao W., Zeng F., Chanieabate M. (2022). Mechanization of Small-Scale Agriculture in China: Lessons for Enhancing Smallholder Access to Agricultural Machinery. Sustainability.

[55] Liu C., Chen L., Li Z., Wu D. (2023). The Impact of Digital Financial Inclusion and Urbanization on Agricultural Mechanization: Evidence from Counties of China. PLoS ONE.

[56] Li Z., Li C., Wang L. (2023). Have Pesticides and Fertilizers Improved Agricultural Development? The Threshold Effect Based on China’s Agricultural Film Usage. Appl. Sci..

[57] Fan M., Shen J., Yuan L., Jiang R., Chen X., Davies W.J., Zhang F. (2012). Improving Crop Productivity and Resource Use Efficiency to Ensure Food Security and Environmental Quality in China. J. Exp. Bot..

[58] West T.O., Marland G. (2002). A Synthesis of Carbon Sequestration, Carbon Emissions, and Net Carbon Flux in Agriculture: Comparing Tillage Practices in the United Stsssssates. Agric. Ecosyst. Environ..

[59] Li Z. (2023). A Review of Research on Cultivated Land Protection Policy. J. Low Carbon Econ..

[60] Li F. (2015). Research on regional food security system from the perspective of production and marketing balance. Jiangsu Social Sci..

[61] Chen L., Peng J. (2024). Influence of high-standard farmland construction policy on grain production capacity and mechanism. Resour. Sci..

[62] Peng J., Zhao Z., Liu D. (2022). Impact of Agricultural Mechanization on Agricultural Production, Income, and Mechanism: Evidence From Hubei Province, China. Front. Environ. Sci..

[63] Lv F., Deng L., Zhang Z., Wang Z., Wu Q., Qiao J. (2022). Multiscale Analysis of Factors Affecting Food Security in China, 1980–2017. Environ. Sci Pollut. Res..

[64] Shi S., Ye Y., Xiao R. (2022). Evaluation of Food Security Based on Remote Sensing Data—Taking Egypt as an Example. Remote Sens..

